# Legal Notes for Institutional Workers

**Published:** 1912-01-13

**Authors:** 


### LEGAL NOTES FOR INSTITUTIONAL WORKERS.

(The Editor will be pleased to answer Legal queries in this column.)

### CAN THE DOCTORS ENFORCE THEIR DEMANDS?

The question whether, by united effort, the medical profession can obtain the concession of the "six cardinal points" is one which is exercising the mind of many a general practitioner at the present time. From the strictly legal point of view it is necessary to bear one important point in mind —namely, that the Insurance Commissioners are already *in esse*, and that this body can alter the text of the Insurance Act itself. They could practically redraft it from end to end if so minded. If the doctors, to a man, refused to act on any Health Committee, the Commissioners (apparently) could enable the Health Committees to act without the assistance of medical members. If no practitioner allowed his name to be inserted on the "panel of doctors," the Star Chamber of National Insurance might decree that insured persons should receive benefit in money instead of in kind. It would therefore seem that no concordat of the medical profession could prevent the administration of the Act in some form or another. If our premisses are correct the doctors are powerless to prevent the impost of new taxes. Small wonder then that institutional managers should look forward with anxiety to the operation of a measure which is certain to decimate their sources of supply.

### APPLYING FOR AN APPOINTMENT.

It is sometimes forgotten by the man who is applying for an appointment that the Committee or other body which has to make a selection is not entirely composed of medical men. Laymen are sometimes found there, and the vote of a single layman may turn the scale. It follows that the *argumentum ad hominem* must not be altogether lost sight of by the candidate who is putting forward his claims to consideration. As it is part of a lawyer's lifework to judge between man and man, a brief note of the points which are likely to impress the mind of him who has to judge between one candidate and another may not be out of place. To begin with, one or two testimonials from those who have had practical experience of a man's work are worth far more than a host of tributes from persons who have not seen the aspirant for a post for the last ten years. Thus, commendation from the senior surgeon at the hospital, who may not be a very well-known man, is more likely to carry weight than a fulsome letter from one of the heads of the profession "who knew you when you were a boy" or who "wants to do a good turn to the son of your father." Again, the candidate must remember that an important post will not be filled merely on letters of recommendation. An interview with the Committee may have to be faced, at which there is sure to be a certain amount of cross-examination. It is worth while to take some trouble to prepare for this ordeal. "Why did you throw up your last appointment? What have you published? What experience have you had of teaching?" These are questions which may have to be answered. Finally (to descend to a very mundane point), although it is said that clothes do not make the man, do not look for a job in your old ones!

### LEGAL POINTS ABOUT TENDERS.

It is customary for those responsible for institutional management to advertise for tenders for the supply of commodities or for the execution of work. The tenders having been received, their relative merits should be carefully considered before any particular one is accepted, because a binding contract springs into existence as soon as the letter of acceptance is *posted*. If acceptance is too long delayed, the advertiser runs the risk of what seems to be the most promising tender being withdrawn. The withdrawal, however, does not take effect until it actually reaches the advertiser; and if he has posted his letter of acceptance before he heard of the withdrawal, there would still be a binding contract. It is scarcely necessary to say that attention should be paid to much else besides the *amount* for which the person tendering offers to supply the goods or to do the work. Nor should the lowest tender necessarily be accepted; and the exercise of a wise discretion in this regard is the more desirable when the object is to erect a new wing or to overhaul an entire drainage system. In carrying out such works difficulties often occur which can be foreseen by the contractor in a large way of business, but are overlooked by a smaller man. Experience is an item for which the big contractor is entitled to charge.

### A KINDLY CUSTOM.

In the course of a case which was recently heard in the King's Bench Division, reliance was placed upon an old-established custom of the medical profession. It was asserted that the practitioner, his wife, and family are entitled to free medical attendance. Without deciding that the custom went so far as this, Mr. Justice Scrutton did hold that there is a custom to attend the widow of a deceased practitioner free of charge. The origin of the practice is lost in the mists of antiquity; but no witness could be called from the ranks of the medical profession to prove that it does not exist. Whether it would be possible to plead this kindly custom in answer to a definite claim for fees is somewhat doubtful; but the silver inkstand which so often adorns the table in the consulting-room bears witness to the fact that in the medical profession "generous friendship no cold medium knows." For binding the members of the profession together; as a means of showing that they are content to set friendship before greed of gain—this unwritten law is of more value than many chapters in the ethical code.

